# La couverture en urgence des pertes de substances cutanées d′origine traumatique de la face antérieure du genou par lambeau fascio-cutané sural à pédicule proximal: à propos de 4 cas de plaies articulaires

**DOI:** 10.11604/pamj.2020.36.58.7321

**Published:** 2020-06-01

**Authors:** Hatim Abid, Ahmed Bouziane, Mohamed El Idrissi, Mohamed Shimi, Abdelhalim El Ibrahimi, Abdelmajid El Mrini

**Affiliations:** 1 Service de Chirurgie Ostéo-articulaire B4, CHU Hassan II, Fès, Maroc

**Keywords:** Mots clés: Genou traumatique, perte de substance cutanée, lambeau fascio-cutané sural, Traumatic knee, loss of cutaneous substance, sural fasciocutaneous flap

## Abstract

Notre expérience porte sur une série de 4 patients, tous de sexe masculin, qui avaient des plaies à la face antérieure du genou avec perte de substance cutanée dans les suites d′un accident de moto, chez lesquels nous avons réalisé au même temps opératoire que le parage, depuis 2012, quatre lambeaux de couverture type fascio-cutané sural à pédicule proximal. L′âge des patients variaient entre 28 et 42 ans. La perte de substance cutanée la plus importante était de 14 x 10 cm. La longueur moyenne du pédicule était de 14 cm. Les suites post opératoires étaient sans complications avec une cicatrisation satisfaisante. Sur le plan fonctionnel le genou et la cheville étaient complètement mobile. Cette étude montre l′efficacité et la fiabilité du lambeau fascio-cutané sural à pédicule proximale dans la couverture des pertes de substances cutanée traumatiques de la face antérieure du genou et permet de limiter les indications des lambeaux musculaires des chefs gastrocnémiens.

## Abstract (en)

Our study reports 4 cases of male patients with wounds on the anterior face of the knee loosing cutaneous substance after motorcycle accident. The patients had undergone surgery involving the debridement and the application of four proximally based sural fasciocutaneous flaps since 2012. The average age of patients ranged from 28 to 42 years. The highest loss of cutaneous substance measured 14 x 10 cm. The average pedicle length was 14 cm. The post-operative suites were uneventful with a satisfactory wound healing. From a functional point of view the knee and the ankle were completely mobile. This study shows the effectiveness and reliability of proximally based sural fasciocutaneous flap in the coverage of post-traumatic losses of cutaneous substances of the anterior face of the knee, and allows for limit the indications for heads of the gastrocnemius muscle flaps.

## Introduction

La perte de substance cutanée au niveau de la face antérieure du genou d′origine traumatique est une entité lésionnelle peu fréquente. Pour la couverture, il existe diverses options dont les lambeaux fascio-cutanés homo-jambier qui fournissent une couverture, une cicatrisation et une fonction très satisfaisantes [1, 2]. Dans la littérature, les travaux qui se sont intéressés au lambeau fascio-cutané sural à pédicule proximale dans la couverture de la face antérieure du genou traumatique sont très limités. Dans ce sens nous rapportons notre expérience à travers une série de 4 cas et mettons le point sur les avantages techniques, esthétiques et fonctionnel de ce lambeau.

## Méthodes

**Figure 1:**
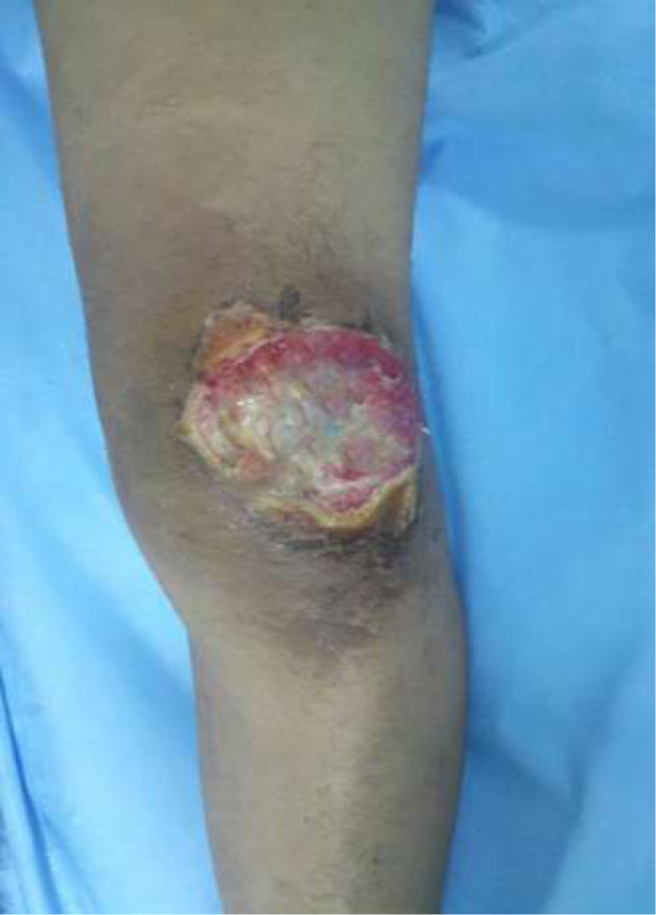
plaie de la face antérieure du genou gauche avec perte de substance cutanée

**Figure 2:**
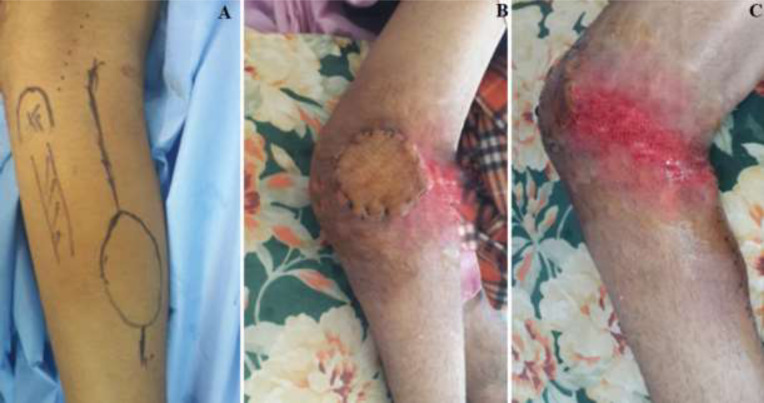
A) dessin au crayon dermographique de la palette cutanée à prélever; B) vue antérieure du lambeau avec une parfaite intégration; C) vue latérale du lambeau avec sa charnière

Il s'agit d'une étude rétrospective, menée dans le Service de Chirurgie Ostéo-articulaire B4 du CHU Hassan II de Fès (Maroc), incluant 4 patients, tous de sexe masculin, opérés dans la période entre janvier 2012 et avril 2015, pour une perte de substance cutanée de la face antérieure du genou post traumatique ayant bénéficié d'un geste de couverture par un lambeau fascio-cutané sural à pédicule proximal. L'âge moyen des patients au moment de la chirurgie étaient de 35 ans. Ils étaient victimes d'une chute de moto, avec une perte de substance cutanée immédiate mettant à nue l'appareil extenseur qui était intact chez tous les malades. Les plaies étaient toutes articulaires par ouverture des ailerons rotuliens (Figure 1). Le bilan radiologique réalisé n'a pas objectivé d'atteinte osseuse. Les patients ont été pris en charge en urgence, dans un délai moyen de 4 heures, par d'abord un parage laissant en place une perte de substance cutanée moyenne de 10 x 10 cm. Puis il a été réalisé dans le même temps opératoire une couverture de l'articulation par un lambeau fascio-cutané sural à pédicule proximale (Figure 2A, Figure 2B et Figure 2C).

## Résultats

Dans notre série, la dimension des pertes de substances cutanées variait de 10 x 10 cm à 14 x 10 cm. Le point de pivotement était à 2 cm en aval du pli poplité. La longueur du pédicule variait de 12 cm à 18 cm. La durée moyenne de l'intervention était de 90 minutes. Tous les sites donneurs étaient fermés par suture simple. Les suites post opératoires étaient sans complications avec une cicatrisation satisfaisante. Sur le plan fonctionnel le genou et la cheville étaient complètement mobiles. Dans tous les cas, la perte de la sensibilité dorso-latérale du pied qui est inévitable était bien tolérée par les patients. Esthétiquement, les malades étaient satisfaits.

## Discussion

Au niveau de la face antérieure du genou, la perte de substance cutanée constitue une véritable urgence chirurgicale, surtout en cas de défect large. Dans la littérature, cette entité lésionnelle a été abordée le plus souvent après arthroplastie du genou, compliquée de désunion post opératoire des points de suture ou de nécrose cutanée secondaire particulièrement dans un cadre infectieux [3-6]. La perte de substance post-traumatique immédiate et massive n'est pas fréquente. Elle représente une lésion hautement grave [7]. Dans ce cadre, Babu *et al*. [8] avaient rapporté en 1994, deux cas de perte de substance post traumatique avec solution de la continuité de l'appareil extenseur. Gaber *et al*. [9] de leur part, avaient rapporté en 2005 une série de 17 patients avec une exposition de la face antérieure du genou par perte de substance cutanée isolée dans 8 cas. En 2009, Jepegnanam *et al*. [10] ont publié un travail similaire qui regroupait 8 patients qui présentaient en plus de la perte de substance cutanée, une rupture du tendon rotulien. Notre série comporte 4 cas de perte de substance cutanée large post traumatique de la face antérieure du genou avec effraction articulaire, survenue par chute de moto chez des malades jeunes, exclusivement de sexe masculin. En pratique, l'unanimité des auteurs accorde la priorité en cas de la mise à nue post traumatique de la face antérieure du genou au parage locale [11]. Ils recommandent de différer le geste de couverture de quelques jours pour mieux choisir le transfert le plus adéquat à la réparation des parties molles. Le délai de couverture ne doit pas dépasser, dans la mesure du possible, une semaine. Ce délai est plus prolongé en cas d'infection ou de nécrose évolutive [10]. La réparation immédiate s'impose en cas de plaie articulaire [7]. Dans notre contexte, les gestes de couverture étaient réalisés en urgence du fait de l'exposition articulaire. Le choix du type du lambeau à réaliser requière une bonne connaissance de l'anatomie vasculaire et des possibilités de transferts tissulaires. Les ressources offertes par l'anatomie ont permis de mettre au point un grand nombre de procédés locorégionaux rendant actuellement le recours aux lambeaux libres exceptionnels.

Dans la littérature, la plupart des travaux concernant la prise en charge des pertes de substance cutanée antérieures et larges du genou rapportent des cas traités par les lambeaux musculaires des gastrocnémiens latéral et médial avec des résultats variables et des complications considérables aussi bien sur le plan esthétique que fonctionnel [12, 13]. L'indication actuelle de ces lambeaux tend, grâce à leur richesse vasculaire, vers l'infection osseuse qu'elle soit sous forme de pseudarthrose septique, d'ostéite ou de sepsis sur prothèse [3-5, 11, 14]. D'un point de vue technique, le plan de clivage du chef médial avec le soléaire n'est pas toujours évident surtout lorsque ce dernier est volumineux. Du coté latéral, le chef musculaire est moins étendu. Le fibula constitue un véritable obstacle au transfert du lambeau et le décroisement du nerf péronier est un geste hautement risqué [7]. De ce fait, le lambeau fascio-cutané sural à pédicule proximal représente techniquement, une option de choix, permettant le prélèvement aisé de palettes cutanées de tailles variables, sensibles, de fine épaisseur, vascularisés par de longs pédicules sans sacrifice vasculaire et avec un arc de rotation qui assure sans difficulté la couverture de la face antérieure du genou. En plus, ce lambeau a l'avantage au niveau du site donneur, de minimiser les séquelles esthétiques inhérentes au prélèvement [7, 11, 13, 15, 16]. Dans la littérature, on ne trouve que peu d'études qui se sont intéressées à ce lambeau dans la couverture des pertes de substances cutanées larges, post traumatiques à la face antérieure du genou. En 1989, Satoh *et al*. [17] ont publié les résultats d'une série de 17 cas traités par le lambeau fascio-cutané sural à pédicule proximal qui permet selon les auteurs de nombreuses possibilités de recouvrement. Dans le travail de Cariou *et al*. [18] de 1995 portant sur 9 cas, les auteurs étaient très satisfaits de la qualité de cicatrisation, de la stabilité et de la sensibilité de la palette cutanée. Ces mêmes constations ressortent dans les publications de Cheon *et al*. [19] en 2008, Suri *et al*. [20] en 2010, et Haitao *et al*. [21] en 2014 qui concluent à l'efficacité du lambeau, à la simplicité des suites post opératoires et à la rareté de complications. Dans notre série limitée en effectif, les résultats esthétiques et fonctionnels très satisfaisants rejoignent la littérature et nous encouragent à la pratique de ce lambeau de plus en plus avec une grande conviction.

## Conclusion

Le lambeau fascio-cutané sural à base proximale est une option thérapeutique fiable et efficace pour la couverture de la face antérieure du genou en cas de perte de substance cutanée post-traumatique. C'est une technique simple et reproductible qui assure de très bons résultats.

### Etat des connaissances actuelles sur le sujet

- La perte de substance cutanée immédiate et massive de la face antérieure du genou d'origine post-traumatique n'est pas fréquente;
- Dans la littérature, la plupart des travaux concernant la prise en charge des pertes de substance cutanée antérieures et larges du genou, rapportent des cas traités par les lambeaux musculaires des gastrocnémiens latéral et médial avec des résultats variables et des complications considérables aussi bien sur le plan esthétique que fonctionnel.

### Contribution de notre étude à la connaissance

- Le lambeau fascio-cutané sural à base proximale est une alternative fiable et efficace pour la couverture de la face antérieure du genou en cas de perte de substance cutanée post-traumatique;
- C'est une technique simple et reproductible qui assure de très bons résultats.
